# Complete Genome Sequence of *Dengue virus* Type 2 from a Resident of North-Central Florida with Locally Transmitted Dengue Fever

**DOI:** 10.1128/genomeA.00782-17

**Published:** 2017-08-03

**Authors:** Sarah K. White, Nicole M. Iovine, L. Connor Nickels, J. Glenn Morris, John A. Lednicky

**Affiliations:** a Department of Environmental and Global Health, College of Public Health and Health Professions, University of Florida, Gainesville, Florida, USA; b Emerging Pathogens Institute, University of Florida, Gainesville, Florida, USA; c Department of Internal Medicine, Division of Infectious Diseases and Global Medicine, University of Florida Health/Shands Hospital, Gainesville, Florida, USA; d Department of Emergency Medicine, University of Florida Health/Shands Hospital, Gainesville, Florida, USA

## Abstract

The majority of dengue fever cases reported in the United States recently have been imported. We isolated dengue virus type 2 (DENV-2) from a North-Central Florida resident with locally acquired dengue fever in May 2016. This is the first evidence of autochthonous transmission of the virus in north-central Florida.

## GENOME ANNOUNCEMENT

*Dengue virus* (DENV) (genus *Flavivirus*, family *Flaviviridae*) is a mosquito-borne virus that is the cause of dengue fever in humans. Five serotypes have been defined (DENV-1 to DENV-5), and while DENV infections may be asymptomatic, 20% of those infected may develop dengue fever, which presents as a fever-rash-arthralgia syndrome (1, 2). Rarely, dengue fever can progress to dengue hemorrhagic fever, which, in turn, can lead to potentially fatal dengue shock syndrome; occurrence of dengue hemorrhagic fever has been associated with sequential infections with different DENV serotypes (1).

When they occur, symptoms of DENV infection are similar to those caused by other arboviruses such as Chikungunya and Zika viruses, posing a challenge for accurate diagnosis (3). Diagnosis of dengue fever is complicated by the limited sensitivity of existing diagnostic tools (4). A majority of the dengue fever cases recorded in the United States in recent years were imported, though DENVs are endemic in Puerto Rico (5). The only available information regarding autochthonous transmission in the continental United States is from outbreaks in Texas during 2005, and in Florida from 2009 to 2011 (6, 7).

On 21 May 2016, a 39-year-old female presented to the University of Florida (UF) Emergency Room with a 4-day history of a sunburn-like rash, shaking chills and severe body aches, and joint pain that precluded walking. The patient reported no travel outside of Florida and had not had sexual intercourse with anyone who had traveled to a known DENV-affected area within the previous 12 months. On day 4 of her illness, saliva, serum, and urine specimens were sent to an arbovirus research laboratory at the UF Emerging Pathogens Institute and screened by real-time reverse transcription (RT)-PCR for Chikungunya, Zika, and DENV-1 to -4 viral genomic RNA (vRNA) (8–10). They tested negative for Chikungunya; Dengue 1, 3, and 4; and Zika vRNAs, but were positive for DENV-2 vRNA. Virus isolation was accomplished by inoculation of aliquots of DENV-2-positive specimens onto MRC-5 and Vero E6 cells, with diffuse cytoplasmic blebbing and apoptosis (as expected with DENV infection) observed in the inoculated cells 15 days post-infection. The vRNA isolated from MRC-5 cells was Sanger-sequenced using PCR primers described by Christenbury et al. (11); a high-fidelity reverse transcriptase and sequence-specific reverse primers for cDNA synthesis; followed by PCR with a high-fidelity thermal polymerase, and 5′ and 3′ RACE performed as previously described (12). The complete virus genome sequence was obtained and designated dengue virus 2 strain Homo sapiens/UF-1/Gainesville/2016. The patient’s muscle and joint pains resolved 5 days after symptom onset.

The complete genome sequence of *Dengue virus* 2 strain Homo sapiens/UF-1/Gainesville/2016 has high identity (99%) with DENV-2 sequences deposited in GenBank from the 2010 to 2011 DENV-2 outbreak in the Peruvian Amazon (GenBank accession numbers KC294203, KC294204, KC294209, and KC294217 to KC294221) and from Haiti in 2014 and 2016 (accession numbers KY415992 and KY702403, respectively), providing clues to the possible origins of the virus.

### Accession number(s).

The complete genome sequence of *Dengue virus* 2 strain Homo sapiens/UF-1/Gainesville/2016 has been deposited in the GenBank database under the accession number KX702404.
